# Post-Intensive Care Syndrome in Survivors from Critical Illness including COVID-19 Patients: A Narrative Review

**DOI:** 10.3390/life12010107

**Published:** 2022-01-12

**Authors:** Charikleia S. Vrettou, Vassiliki Mantziou, Alice G. Vassiliou, Stylianos E. Orfanos, Anastasia Kotanidou, Ioanna Dimopoulou

**Affiliations:** First Department of Critical Care Medicine & Pulmonary Services, School of Medicine, National and Kapodistrian University of Athens, “Evangelismos” Hospital, 106 76 Athens, Greece; mantziouv@yahoo.gr (V.M.); alvass@med.uoa.gr (A.G.V.); sorfanos@med.uoa.gr (S.E.O.); akotanid@med.uoa.gr (A.K.)

**Keywords:** quality of life, depression, anxiety, post-traumatic stress disorder, post-intensive care syndrome, COVID-19

## Abstract

Current achievements in medical science and technological advancements in intensive care medicine have allowed better support of critically ill patients in intensive care units (ICUs) and have increased survival probability. Post-intensive care syndrome (PICS) is a relatively new term introduced almost 10 years ago, defined as “new or worsening impairments in physical, cognitive, or mental health status arising after critical illness and persisting beyond acute care hospitalization”. A significant percentage of critically ill patients suffer from PICS for a prolonged period of time, with physical problems being the most common. The exact prevalence of PICS is unknown, and many risk factors have been described well. Coronavirus disease 2019 (COVID-19) survivors seem to be at especially high risk for developing PICS. The families of ICU survivors can also be affected as a response to the stress suffered during the critical illness of their kin. This separate entity is described as PICS family (PICS-F). A multidisciplinary approach is warranted for the treatment of PICS, involving healthcare professionals, clinicians, and scientists from different areas. Improving outcomes is both challenging and imperative for the critical care community. The review of the relevant literature and the study of the physical, cognitive, and mental sequelae could lead to the prevention and timely management of PICS and the subsequent improvement of the quality of life for ICU survivors.

## 1. Introduction

The consequences of critical illness can have a prolonged effect on intensive care unit (ICU) survivors, involving their physical, psychological and cognitive health. For the constellation of these symptoms, the term “post-intensive care syndrome” (PICS) has been used in the literature and is nowadays accepted that it can have detrimental effects on patients’ lives, and particularly on health-related quality of life (HRQOL). In the medical literature, as well as by most clinicians, the term “PICS” is used to describe the “new or worsening impairments in physical, cognitive, or mental health status arising after critical illness and persisting beyond acute care hospitalization” [1]. This definition includes patients who live in rehabilitation facilities, specialized nursing units, or at home. It does not include, however, patients who were admitted to the ICU with primary neural injuries, such as traumatic brain injury or cerebrovascular accidents.

Although there is no time frame for PICS and it can last for a prolonged period of time [2], it is usually described during the time period following ICU discharge. The risk factors for the emergence of PICS are not clearly defined and vary in different studies, however they are generally separated into two categories: those that refer to pre-existing factors, such as neuromuscular or neurological disease, and other severe comorbidities or psychiatric history, and those that are related to the ICU, including the presence of delirium, the dose of administered sedatives, and the presence of acute respiratory distress syndrome (ARDS), sepsis, or dysglycemia. The family and proxies of ICU survivors can also be affected, particularly regarding their psychological health, as a response to the stress they suffered during the critical illness of their kin. This separate entity is described as PICS family (PICS-F) [3].

It is not feasible to estimate with accuracy the frequency of PICS, partly due to the lack of consensus on the assessment tools used for its diagnosis and evaluation (Table 1), yet it has been reported that more than half of ICU survivors will present at least one symptom from the cognitive, physical, or psychological sphere [4,5,6,7]. A particular challenge of the modern era is the PICS syndrome related to the coronavirus disease 2019 (COVID-19) pandemic due to the large number of patients admitted to ICUs, the pressure exerted on the healthcare systems and rehabilitation facilities, and the extreme conditions of hospital and social isolation that COVID-19 patients and families experienced, which were unprecedented.

In this narrative review, we are aiming at presenting the current knowledge on the physical, cognitive, and psychological components of PICS. We will describe the modern concepts of its pathophysiology, prevention, and management, including the new concept of “post-COVID-19 PICS”.

## 2. Physical Dysfunction

**Figure 1:**
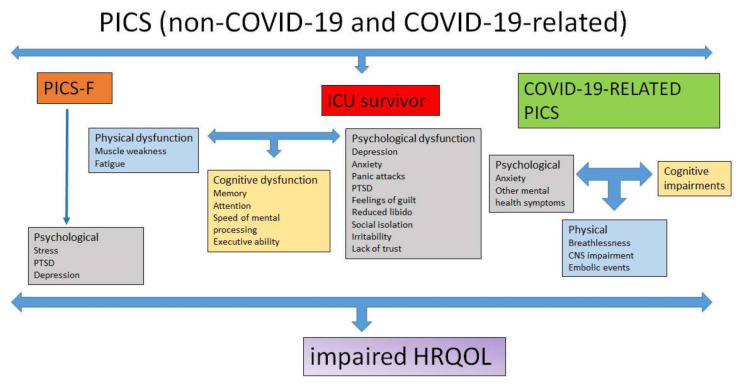
Commonest symptoms that characterize the post-intensive care syndrome in non-COVID-19 and COVID-19 patients, and their effect on HRQOL. COVID-19, Coronavirus disease 2019; HRQOL, Health-related quality of life; ICU, Intensive care unit; PICS, Post-intensive care syndrome; PICS-F, PICS family; PTSD, Post-traumatic stress disorder.

Physical impairment after critical illness is a recognized part of PICS, affecting about one-third of ICU survivors, yet the underlying pathophysiological mechanisms remain poorly understood. The symptoms include muscle weakness, fatigue, sleep disturbance, weight loss, respiratory dysfunction, and dysphagia [39]. Physical symptoms can cause persistent impairment affecting daily life activities, such as performing household chores and taking medications, thereby seriously affecting HRQOL (Figure 1) [1]. Table 1 lists, in brief, the different assessment tools used in the evaluation of physical dysfunction.

The diagnosis of PICS-related muscle weakness is usually made with the use of the Medical Research Council scale, in which the strength of both upper and lower extremities is graded from zero (no movement) to five (normal power) [40]. Although the incidence of muscle weakness is high, reported to be around 40% [41], its pathophysiological mechanisms are not completely elucidated, and the current understanding of this entity is regarded to be multifactorial [39]. In a murine model of PICS following induced sepsis, the animals that survived exhibited reduced total mileage at the treadmill test [39]. Factors that may contribute to deteriorating muscle function in critically ill humans, according to current theories, include the prolonged catabolic state and bed rest induced by stress and critical illness, and the evolution of ischemia in the microvascular level of the muscle and supplying nerves that can damage cellular ion channels and mitochondria [42]. Although muscle weakness may resolve after several weeks to months, in numerous cases, the impairment of motor function persists for months to years [43,44]. The presence of joint contractures and/or ectopic ossifications may actually further worsen motor function and HRQOL [45,46].

Apart from experimental models, prospective studies have also described risk factors, including female sex, sepsis, catabolic state, multiorgan failure, systemic inflammatory response syndrome, long duration of mechanical ventilation, immobility, hyperglycemia, and the administration of glucocorticoids and neuromuscular blocking agents [40,42,47,48,49]. Other risk factors include ARDS, older age, hyperoxia, and vasopressor administration [13,48,49,50,51,52,53,54,55]. The relationship between the administration of paralytic agents and muscle weakness has not been reproduced; however, a systematic review showed that a correlation may exist in patients with sepsis [40,48,56]. Co-existing sleep disturbance, which is present in 50–66% of patients, might also play a role in the reported weakness by increasing fatigue (Figure 1) [57].

Critically ill COVID-19 survivors are likely at increased risk for chronic pain, which can further affect rehabilitation and recovery [58]. According to current guidelines, COVID-19 patients with severe symptoms are treated with steroids. Steroid use is known to cause significant side effects, namely immune dysfunction, dysglycemia, frail skin, osteoporosis, sarcopenia, loss of muscle mass, nervousness, and changes in mood [59].

Muscle strength was also independently correlated with mental status and HRQOL [60], while muscle weakness seemed to contribute to the cognitive and mental sequelae in PICS (Figure 1). There is no therapeutic intervention of proven efficacy for PICS-related muscle weakness; however, several interventions have been assessed for prevention and symptom management and are under investigation [51,61,62].

## 3. Cognitive Dysfunction

**Figure 2:**
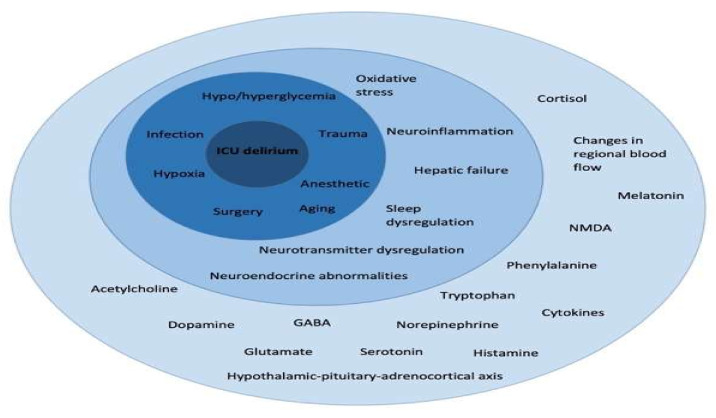
ICU delirium has a complex and multifactorial pathophysiology at the cellular and molecular level that affects the clinical level with significant overlap. This complexity explains the observed difficulty in delirium treatment and highlights the importance of prevention. GABA, Gamma-aminobutyric acid; ICU, Intensive care unit; NMDA, N-methyl-D-aspartate.

The term “cognitive dysfunction” refers to persistent defects in brain function, combined with behavioral and emotional changes, that result in the inability to function normally in everyday life and subsequently low HRQOL. Patients with cognitive dysfunction often present with problems in memory, attention, speed of mental processing, speaking and executive ability, with the latter including organization, design, and problem-solving. Some authors make a distinction between “cognitive dysfunction” and “cognitive impairment”, with the term “impairment” referring to a more permanent condition, while “dysfunction” implies an acute state that can change or improve [63]. Cognitive impairment in ICU survivors can be caused by numerous physiological and biochemical factors (Figure 2) [64]. Frequencies also vary in different studies, mainly due to the different assessment tools used and the variable timings of assessment (Table 1), ranging from 20–60 % [65,66,67], while symptom duration was found to extend for up to 8 years [5,50].

It has been supported that patients with comorbidities such as vascular disease, diabetes, chronic obstructive pulmonary disease, human immunodeficiency virus infection, and pre-existing cognitive impairment may be particularly vulnerable to neurological ICU complications [68]. It is likely that people with pre-existing cognitive dysfunction, such as mild Alzheimer’s disease characterized by chronic cognitive decline, can deteriorate further after neurological insults similar to those during ICU stay.

### 3.1. Risk Factors for Cognitive Decline in ICU Survivors

Risk factors can be categorized into two groups, modifiable and non-modifiable. Modifiable factors include delirium during ICU stay (Figure 2), the application and length of mechanical ventilation, the presence of hypoxia and dysglycemia, the use of psychotropic medications, blood pressure derangements, and transfusion with blood and blood products. The non-modifiable factors include age, comorbidities, education level, pre-existing cognitive dysfunction, e.g., dementia, the presence of the apolipoprotein E allele, and the severity of illness [69,70]. ICU delirium, which is a multifactorial condition with complex pathophysiology, is the best-studied risk factor in surgical and general ICU populations, and a relationship between the length of delirium and cognitive decline has been described in ARDS [71]. COVID-19 patients are at increased risk of developing ICU delirium due to invasion of the central nervous system from the virus, the inflammatory storm syndrome that is accompanied by encephalopathy, and the severity of multiple organ failure also affecting the brain [72,73,74]. Cognitive decline following ARDS is more pronounced in older patients with pre-existing dysfunction. The BRAIN-ICU study showed that a longer ICU stay was related to poorer results in cognitive function tests at three and twelve months after ICU discharge in a mixed group of patients [1]. Survivors who complained of memory impairment also reported anxiety, depression, and PTSD [75], and scored lower in HRQOL questionnaires up to one year after hospital discharge. A lower performance in cognitive function tests was related to low Informant Questionnaire on Cognitive Decline in the Elderly (IQCODE) score, low Acute Physiology And Chronic Health Evaluation II (APACHE II) score at the time of ICU admission, high serum Neuron Specific Enolase (NSE), interferon (INF)-γ levels, longer time from admission to the first dose of antibiotics, administration of haloperidol, and higher glucose levels, while higher education was related to better cognitive outcomes [76].

### 3.2. Persistence of Cognitive Impairment

For many ICU survivors, there is a significant improvement in cognitive function one year after hospital discharge. However, for ARDS survivors, cognitive dysfunction can be persistent, can affect the ability to work, while a subset of this population will not improve significantly [28,75,77]. A different pattern has been described for cognitive function in sepsis and septic shock patients. Sepsis-induced cognitive dysfunction improved with time and was related to ICU length of stay, level of education, cognitive reserve, glycemia control, and NSE levels [76]. In a preliminary study published in 2013, Semmler et al. [78] studied morphology, electrophysiology, neurology, and behavior of ICU survivors with and without sepsis, and normal controls. The authors reported deficits in verbal learning and memory and a reduction in the volume of the left hippocampal area in septic patients compared to controls. Non-septic ICU survivors, on the other hand, showed electrophysiological findings suggestive of unspecific brain dysfunction [78].

## 4. Psychological Dysfunction

Psychological sequelae as a result of critical illness and ICU admission are very common and can have a significant effect on HRQOL; therefore, each patient with suspected PICS should undergo a psychological assessment [1,79,80]. The most common psychological problems encountered are depression, anxiety, panic attacks, PTSD, feelings of guilt, reduced libido, social isolation, irritability, and lack of trust (Figure 1) [81]. These psychological problems are usually accompanied by fatigue, loss of interest, loss of appetite, feelings of despair, disturbed sleep, and sexual dysfunction [82,83]. Patients may find challenging to complete even simple everyday tasks [84]. Disease, per se, its complications, prolonged bed rest, medication side effects, medical and nursing procedures performed, and limited physical function and autonomy lead to impaired physical ability that also affects mood and mental health. Depression and anxiety, therefore, follow ICU discharge [85,86]. Psychiatric symptoms may be due to psychological response to physical or psychological stress, to brain injury caused by the disease or the imposed treatment, or both [87]. Medications, physiological changes, pain, altered sensations, and a new and unknown environment are potential factors for the cause of psychological sequelae. Table 1 shows in brief the various assessment tools used in the psychological evaluation of patients with PICS.

Patients admitted to the ICU with COVID-19 experience additional stress resulting from physical isolation and distancing from relatives, friends, and healthcare professionals due to strict preventive measures and extensive use of personal protective equipment [88].

### 4.1. Depression

Depression symptoms are important for ICU survivors. Their recognition is paramount since their presence has been linked to prolonged abstain from work, decreased HRQOL, and suicide risk [89,90]. Potential pathogenetic mechanisms of depression and anxiety in ICU survivors involve organ dysfunction, medications, pain, lack of sleep, increased cytokine levels, stress-related activation of the hypothalamic-pituitary axis, hypoxemia, and brain injury-induced neurotransmitter dysfunction [89,90]. Depression occurs in 25–60% of survivors of critical illness [91,92,93]. A significant association between post-ICU depressive symptoms measured at hospital discharge and female sex has been described [38,94,95]. It has also been documented that depressed mood in the month prior to ICU admission could predict depressive symptoms up to 2- and 6-months post-ICU, as could poor pre-ICU physical functioning [38,95]. There are several studies that have examined the association between ICU treatment and depression. ICU length of stay and severity of illness at ICU admission, as measured by the APACHE II score, were not significant predictors for depressive symptoms [94,96]. Studies have also examined the predictive ability of early post-ICU memories of in-ICU experiences in depressive symptoms [94]. One study found that poor recollection of the ICU period at hospital discharge, however not memories of frightening experiences, could predict depressive symptoms at 6 months [94]. Stressful memories and nightmares while in the ICU or a sense of fear 5 days post-discharge could predict depressive symptoms later in life [96,97].

### 4.2. Anxiety

Anxiety is the least studied symptom in ICU survivors. It is related to other psychiatric symptoms, memories and delusions, while patients with anxiety also report excess unrest, sensitivity, and fatigue [94,97,98,99,100,101]. In ICU survivors, the reported frequencies for anxiety range from 16–62%, however different tools for assessing symptoms have been used (Table 1) at different time points post-discharge [100,102,103,104,105]. There was no difference in anxiety frequency between medical or surgical patients or patients with trauma [106]. Anxiety symptoms seem to persist from 3 to 14 months after ICU discharge [106]. No correlation has been shown between anxiety and age, sex, disease severity, or length of ICU stay [107].

### 4.3. Post-Traumatic Stress Disorder (PTSD)

PTSD’s main characteristic is the exposure of a subject to an event that is life-threatening or perceived as such. Following this traumatic experience, patients present with intrusive thoughts, avoidant behavior, general irritability or paranoia, and other hyperarousal symptoms, reduced cognition involving the inability to concentrate on one thing, and mood disturbance. The association between PTSD and critical illness remains unclear, while prevalence estimates vary significantly from 4 to 62% of ICU survivors [106,108,109]. A systematic review reported PTSD to be present for up to eight years in 24% of the studied population. No correlation was found between disease severity and PTSD [99]. A multicenter study from Britain, including 26 ICUs, reported that PTSD followed anxiety and rarely occurred alone [37]. Risk factors included traumatic memories during ICU stay, duration of sedation, opioid dosage, nightmares, and feeling breathless [99,110,111]. Delirium in the ICU and benzodiazepine dose have also been reported to be related to PTSD [112]. Other risk factors are pre-existing depression and anxiety disorder [106], lower education level, alcohol abuse, and female sex [99]. Anxiety prior to PTSD has been described as a risk factor for PTSD in the general population [113].

## 5. PICS Family

Apart from ICU survivors, their families also suffer from PICS. This is called by experts PICS family (PICS-F), and is defined by the presence of new physical or psychological symptoms in the relatives of patients treated and discharged from the ICU (Figure 1) [2,114]. The symptoms may persist for months or even up to 8 years, particularly if the patient dies or is discharged with severe disability. The frequency of the problem varies greatly in different studies, and this wide range is explained by the different methodologies applied regarding both timing and assessment tools (Table 1). The most commonly reported symptoms affecting mental health are anxiety, depression, PTSD and/or complicated grief, which may eventually lead to compromised quality of life, and even to the loss of employment months (Figure 1) [115,116,117,118,119,120,121]. Anxiety symptoms were present in at least half of the family members in a time frame of six months [115,116,117,118,119], while depression symptoms were also common, yet less frequent over time [118,119,120,121].

The causes and factors that increase the risk of PICS-F include patient and family characteristics and ICU characteristics. Older age, insufficient information regarding ICU care, prolonged ICU stay, poor communication with ICU staff, visiting restrictions, low income, increased financial burden from caring for patients, previous history of anxiety and depression, particularly requiring medication, and a history of personal ICU experience, are some of the predisposing factors [114,115,116,122,123,124,125].

## 6. COVID-19-Related PICS

**Figure 3:**
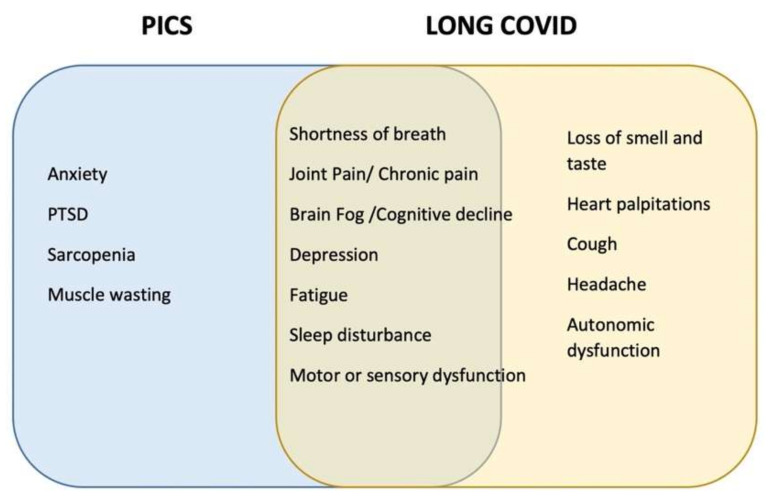
Post-intensive care syndrome (PICS) (blue rectangle) and “Long COVID” (yellow rectangle) overlap. The two conditions present with many symptoms that show significant overlap shown in the middle green section. COVID, Coronavirus disease; PICS, Post-intensive care syndrome; PTSD, Post-traumatic stress disorder.

COVID-19 is an acute viral infection, and most cases are asymptomatic or present with mild symptoms. However, a subset of patients may develop respiratory failure, or even ARDS, requiring mechanical ventilation, with a mortality rate in the range of 20–40% [126]. Survivors of critical illness related to COVID-19 are at high risk of developing PICS for a number of reasons [73,74,88]. Severe acute respiratory distress coronavirus 2 (SARS-CoV-2) may have long-lasting symptoms, persisting long after ICU discharge, also described as “long COVID”, which show significant overlap with PICS, and may exacerbate its symptoms (Figure 3) [127,128,129].

There are already expressed fears that post-COVID-19 PICS will overwhelm healthcare facilities designed and available for post-ICU rehabilitation [130]. Multiple centers are creating multidisciplinary programs for the post-COVID-19 population [131,132]. Authors report that the physical, psychological and cognitive impairments observed in post-COVID-19 ICU survivors were comparable to those observed in non-COVID-19 patients [133]. The most frequently reported symptoms were neurological symptoms, anxiety, breathlessness, malnutrition, dietary insufficiencies, and embolic events [134]. When HRQOL was assessed in a case series of post-ICU COVID-19 cases, they all showed impairment when assessed with the use of the SF 36 at 3 months [135]. A call has already been made to the World Health Organization (WHO) to develop International Classification of Disease Diagnostic Codes for PICS in the “Age of COVID-19” [136]. Follow-ups of these patients in post-ICU clinics and in primary care are paramount and, in many cases, must be performed with virtual visits, and support of patients and families must be provided through the internet [137]. PICS-F can also be aggravated during the pandemic due to distancing from loved ones during hospitalization, loss of social support, and inability to “say goodbye” if bereaved [138].

The mental health of healthcare providers has also been significantly affected by the pandemic. Even when healthcare workers were performing their duties without any psychological issues expressed, they were still exposed to increased risk for anxiety and depression, particularly those working in the frontline and treating patients with COVID-19. The main factors contributing to this increased risk are reported to be the increasing number of cases requiring treatment, leading to overwhelming workload, increased patient mortality, and shortages of personal protective equipment during the pandemic. Other significant factors include fear of contracting COVID-19 and fear of infecting others, particularly family members, as well as social stigmatization and discrimination [139]. Lack of feeling of personal accomplishment and feelings of depersonalization were also common amongst healthcare workers, while emotional exhaustion was an independent predictor of PTSD [140].

## 7. PICS Prevention and Treatment

**Figure 4:**
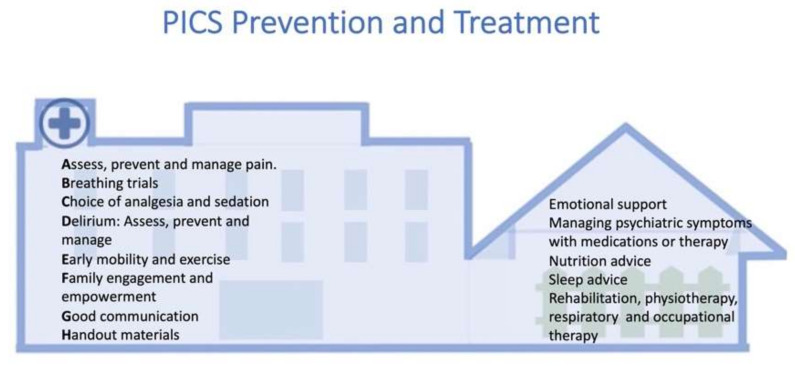
Post-intensive care syndrome (PICS) prevention and treatment. The ABCDEFGH bundle is a bundle of interventions that can assist in PICS and PICS family (PICS-F) prevention and is mainly instituted during intensive care unit (ICU) stay, while PICS treatment is extended after ICU discharge at the rehabilitation facilities or when the patient returns home and can be supported by specialized follow-up clinics.

Several interventions have been proposed for PICS prevention. Recent guidelines can be summarized in the “ABCDEFGH” bundle and aim to prevent long-term cognitive impairment, delirium, and physical decline in the ICU [141]. The bundle is shown in Figure 4 and has the following components: (A) refers to assessing and managing pain by using validated tools. It is important to consider both pharmacological and non-pharmacological interventions for pain management. (B) refers to allowing spontaneous awakening and breathing trials, unless contraindicated, and identifying and correcting communication barriers for the patient. (C) refers to the choice of sedative, avoiding benzodiazepine-based sedation, and administering the least quantity of sedatives possible. (D) refers to daily delirium monitoring, combined with pharmacological and non-pharmacological measures for prevention and treatment. (E) refers to early mobility assessment and physiotherapy that reduces physical decline, loss of muscle mass, and duration of delirium. Neuromuscular electrical stimulation is a physiotherapy-related additional promising modality [142,143,144]. (F) refers to family engagement and empowerment by using standard communication strategies and also involving social workers and other team members. (G) emphasizes the need for good communication practices, particularly for preventing and ameliorating PICS-F, and (H) refers to handout material that can be provided to carers and families [141]. Supportive measures additional to the bundle include avoiding hypoglycemia and hypoxemia during ICU stay, which are linked to the presence of encephalopathy and delirium, access to cellphones and tablets for direct communication, the use of ICU diaries that can be read by the family members, and support groups with family members and friends of patients, which can also ameliorate the symptoms of PICS-F [70,145,146,147]. The use of the “ABCDEFGH” bundle alone or in combination with additional measures may lead to a three-fold increase in patients returning to independent functioning after hospital discharge [141].

Even when PICS is established, there are still interventions that may promote recovery. Post-ICU clinics that provide follow-up to ICU survivors can be useful by providing counseling and support to patients and their families, as well as proper education about rehabilitation, good nutritional status, and adequate sleep [148]. A multidisciplinary approach seems reasonable given the multifactorial nature of PICS; however, there are several burdens to its application. Lack of organization, adequate resources, appropriate staff, and high-quality evidence for its effectiveness renders the treatment of PICS a very difficult endeavor, highlighting the need for prevention and early recognition. Regarding the need for acquiring high-quality evidence, there is ongoing research aimed at establishing clear definitions, methods, and assessment tools that will standardize the diagnosis and enable the production of generalizable evidence.

In a recent consensus statement [9], a set of instruments for the assessment of PICS was proposed, which could support the evolution of PICS research. This set incorporates several of the scales shown in Table 1 that have been validated for ICU populations. Such scales are Patient Health Questionnaire-4 and 8 (PHQ-4 and PHQ8) [32,36], Generalized Anxiety Disorder Scale-7 (GAD-7 [32] and Impact of Event Scale–Revised (IES-R) [149] for mental health, the MiniCog, [23] Animal Naming [24], Repeatable Battery for the Assessment of Neuropsychological Status (RBANS) [25], the Trail Making Tests (TMT) A and B [26] for the assessment of cognition, and the Timed Up-and-Go (TUG) [8], the handgrip strength [10], the 2-Minute Walk Test (2-MWT) [11], and the Short Physical Performance Battery (SPPB) [12] for the assessment of physical function. The European Quality of Life 5 Dimensions 5 Level (EQ-5D-5L) and the 12-Item WHO Disability Assessment Schedule (WHODAS 2.0) have been proposed for the assessment of HRQOL for patients and caretakers, respectively [16].

Patients with psychiatric symptoms may benefit from treatment with a combination of pharmacotherapy and non-pharmacological measures, such as psychological and behavioral therapies [150]. There is not a specific medication that has been proven to be beneficial in PICS-related psychiatric symptoms, and it is not clear whether PICS-related psychiatric problems differ pathophysiologically from depression, anxiety, and PTSD not related to PICS. A recent report in a murine PICS model suggested that the GABAergic system, and in particular the parvalbumin-related interneuron dysfunction in the hippocampus, may play a significant role in PICS pathophysiology, and that the early treatment with fluoxetine, an antidepressant agent, can alleviate this pathology [151]. Relevant results from human studies are still lacking. Regarding other therapies, cognitive-behavioral therapy (CBT) is considered the first-line non-pharmacological treatment for most of the psychological entities of PICS and PICS-F, namely depression, anxiety, and PTSD [152]. CBT programs have the advantage of being applied via smartphones and computer applications, and can therefore be delivered under the strict conditions of the pandemic [153]. Post-ICU clinics may again provide the necessary link for the timely recognition of cognitive or other psychiatric PICS symptoms.

Physical dysfunction requires a multidisciplinary approach that includes exercise, physiotherapy, occupational therapy, and rehabilitation and requires the involvement of critical care physicians, neuropsychiatrists, physiotherapists, and respiratory therapists (Figure 4) [154]. New promising techniques that target recovery from PICS by combining objective lean body mass and metabolic assessments, novel nutrition and exercise interventions can be applied in this setting [53,60,61]. For example, it has been shown that muscle glycogen stores are depleted early during critical illness [60]. This is important since muscle protein must then be broken down by the body for energy once glycogen stores are depleted. New ultrasound technologies have been developed that measure muscle glycogen content, and when combined with appropriate nutritional interventions, may assist in restoring anabolic ability and reducing or reversing muscle loss [155]. Similar approaches aimed at personalized medicine also involve the design of appropriate personalized rehabilitation plans that combine aerobic and workload exercise with electrical muscle stimulation [60,142].

## 8. Conclusions

Admission to the ICU, even if the outcome is positive, often bears significant sequelae for the patients and their carers and family. PICS involves physical, cognitive, or mental health problems and affects more than half of ICU survivors worldwide. Numerous studies, including meta-analyses, have reported poor HRQOL related to PICS in ICU survivors in the months and years following hospital discharge. PICS-F refers to psychological symptoms that present to the families and carers of ICU survivors, and these symptoms may be comparable in severity to the symptoms of the patients. Despite the importance of PICS, there are many knowledge gaps in our understanding of its pathophysiology and, consequently, in our strategies for prevention and treatment. The emergence of the COVID-19 pandemic has aggravated the problem, with an increased number of patients in need of ICU, and post-ICU care, under unprecedented circumstances of demand and social distancing. While technological innovation and the expanding use of telecommunication are recruited in order to face the current challenges, collective action and a greater consensus on related definitions and assessment tools are prerequisites for the design of prospective clinical studies, and for the generalization and application of their results in the future. Experimental studies can also contribute to expanding our knowledge, particularly on the physical and cognitive components of PICS.

## Figures and Tables

**Table 1:** Tools used for the assessment of mental health, cognition, physical function, and quality of life in patients with post-intensive care syndrome (PICS).

Physical Function
Timed Up-and-Go (TUG) [8,9]
Handgrip strength [10]
2-Minute Walk Test (2-MWT) [11]
Short Physical Performance Battery (SPPB) [12]
Comprehensive Geriatric Assessment (CGA) [13]
Medical Research Council scale for muscle strength (MRC) [14]
**Health-Related Quality of Life (HRQOL)/Subjective Health**
European Quality of Life 5 Dimensions 5 Level (EQ-5D-5L) [15]
Subjective concern [9]
WHO Disability Assessment Schedule (WHODAS) 2.0 [16]
World Health Organization Quality of Life (WHOQOL)-100 and (WHOQOL)-Bref [17]
RAND corporation tool for HRQoL (RAND 36) [18]
36-Item Short Form Survey (SF-36) [19]
Activities of Daily Living and Instrumental Activities of Daily Living (ADLs and IADLs) [20]
Functional Independence Measure [21]
Healthy Aging Brain Care Monitor Self Report version (HABC-M SR) [22]
**Cognition**
MiniCog [23]
Animal Naming [24]
Repeatable Battery for the Assessment of Neuropsychological Status (RBANS) [25]
Trail Making Test (TMT) A, B [26]
Mini-Mental State Exam (MMSE) [27]
Wechsler Adult Intelligence Test-Revised [28]
Wechsler Memory Scale-Revised [28]
Rey Auditory-Verbal Learning Test [28]
Rey–Osterrieth Complex Figure Test [28]
Oklahoma Premorbid Intelligence Estimation method (OPIE) [28]
Verbal Fluency test [28]
Logical memory, Visual Reproduction, and Adult Video Learning Test (AVLT) [29]
Short Test of Mental Status, Modified Hachinski Scale, Prime MD [29]
Picture Completion, Block Design [29]
Boston Naming Test, Category Fluency [29]
**Mental Health**
*Depression*
Center for Epidemiologic Studies Depression (CES-D) [30]
Beck Depression Inventory [28]
Geriatric Depression Rating Scale-Short Form (GDS-SF) [31]
*Anxiety*
Generalized Anxiety Disorder Scale (GAD) [32]
Beck Anxiety Inventory [28]
State-Trait Anxiety Inventory (STAI) [33]
*Post-Traumatic Stress Disorder*
Impact of Event Scale—revised (IES-R) [34]
Post-Traumatic Stress Disorder Checklist for a Specific event (PCL-S) [28]
Post-Traumatic Stress Disorder Check List—Civilian version (PCL-C) [28]
Clinician-Administered Post-Traumatic Stress Disorder Scale (CAPS) [35]
Davidson Trauma Scale (DTS) [35]
Posttraumatic Stress Diagnostic Scale (PDS) [35]
Post-Traumatic Stress Syndrome—Question Inventory (PTSS) [35]
*Global*
Patient Health Questionnaire (PHQ)—various versions [32,36]
Depression Anxiety and Stress Scales instrument (DASS-21) [28]
Hospital Anxiety and Depression Scale (HADS) [37]
Structured Clinical Interview for DSM-IV (SCID) [38]

## Data Availability

Not applicable.
